# Civilization and Tuberculosis

**Published:** 1896-12

**Authors:** F. Paschal

**Affiliations:** San Antonio, Texas


For the Texas Medical Journal.

### CIVILIZATION AND TUBERCULOSIS.

BY F. PASCHAL, M. D., SAN ANTONIO, TEXAS.

[Read at the Annual Meeting of the West Texas Medical Association, held in San Antonio, Texas, October, 1896.]

IF TUBERCULOSIS is an infectious disease, then it is preventable; and civilization is not giving it the attention its importance demands. Federal, State, municipal, and home governments should, by concerted and intelligent action, adopt measures to prevent the enormous destruction of life that is daily caused by tuberculosis. How to do this could be determined by study, and experience. Many cities have placed tuberculosis on the list of contagious diseases made returnable to the health officers, but it is impossible to say where this is done, whether it has diminished the mortality of the disease; because it has not been in operation long enough, or thoroughly enough to determine.

Any other infectious disease known to cause so large a proportion of the deathc from all causes (one death in every seven deaths), would stir any other nation to its fullest endeavors to protect human life, and yet State, Federal, and municipal governments remain passive to an infectious disease that is the cause of more deaths than the combined, infectious, zymotic, and contagious, diseases. Quarantine is immediately declared if yellow fever, small-pox, or cholera heralds its coming, and no expense is spared to prevent these diseases. This is right; for above all other earthly considerations health and life must rank first. Does it seem impossible that tuberculosis, which is acknowledged to have its source of contagion in sputa, and food, cannot be prevented, and that it must continue to propagate itself and endanger every family in this country? I believe not; and look for the day to come when, like yellow fever, cholera, and small-pox, consumption will be an infrequent disease.

I do not advocate a general quarantine against tuberculosis, as this would be impossible. No one is endangered by associating with the tuberculous if ordinary care is used to prevent contagion. I do, however, advocate placing the disease on the list of contagious diseases, and making it returnable to health officers, that the needed precautions may be adopted.

There is one measure that deserves attention;—that is, the proper care of consumptives who are dependent upon charity for protection. We all know that when misfortune falls upon mankind, and one must ask of municipal or county boards food and shelter, that he then becomes a tax upon every taxpayer in city and county, and there is no tax that is more cheerfully paid than that which is paid for charity, and it is but just to the taxpayers and the recipients of charity, alike, that every comfort and care should be given them. Are crowded poor houses and city hospitals fit places for consumptives? Does either institution offer prospects of relief or cure, or prevent the spread of this disease? Certainly not. Therefore, if these cases are our wards, and we must care for them, why not do so in the most humane way? Let the States levy a tax for this purpose, and build institutions in suitable places for those so unfortunate as to have neither strength, means, relatives nor friends. Here they could have every comfort and care possible to give them, and which they are justly entitled to receive; and in this way thousands of cases could be isolated, and a better opportunity afforded them for relief.

Indeed, I cannot see why such institutions should not be built by the Federal government in the healthiest localities in this country, and those cases that are confined in bleak poor houses and crowded hospitals sent there. Legislation could devise the means of support.

It might be argued that this would be an infringement on the constitutional liberties of man; but when dealing with those who, from necessity, surrender themselves to the care of poor houses and city hospitals, humanity should rank first, and they should be cared for as our advanced civilization demands, and the infectious nature of the disease requires.

Another point worthy of consideration is, that while tuberculosis is not believed to be transmitted, but that those born of tuberculous parents acquire this disease far more readily than those who are not born of tuberculous parents, therefore children of tuberculous parents have less powers of resistance to this disease. Parents who have children with tuberculous tendencies should pay particular attention to prevent anything being done to undermine their health, and should do everything possible to develop their strength. Children must be educated; children of tuberculous parents as well as others, but while being educated, there is no question that in many instances the constitution of these children is undermined, and the foundation laid to acquire the disease. Crowded, illy-ventilated school rooms, long hours, hard study, cold lunches, etc., is not the way to make delicate children strong. Even the healthiest-looking children, at the beginning of the long school term, look pale and faded at the end of it. Let them be educated, but do not task all alike. Let delicate children have shorter hours, healthy exercise, and wholesome food, and there would be less catarrh of the stomach, more strength and vitality; their resisting power would be greater, with better physical development.

It is impossible to count the childless homes caused by parents anxious for their children to be prepared for life's battles, and on whom the blight of an early grave is marked.

Are we, as physicians, doing our part to prevent this disease? Unquestionably we are not. We watch the sunken eye, the hollow cheek, the quick breath, the emaciated form, and hear the hollow cough and feeble voice, and feel and know how utterly useless all of our efferts are to relieve them, and yet very few of us urge parents to care for their children, cities to protect her citizens, or governments to help stamp out a *preventable disease.*

